# Experimental Study on Shear Performance of Cast-In-Place Ultra-High Performance Concrete Structures

**DOI:** 10.3390/ma12193254

**Published:** 2019-10-05

**Authors:** Chuanxi Li, Zheng Feng, Lu Ke, Rensheng Pan, Jie Nie

**Affiliations:** 1 Key Laboratary of Bridge Engineering Safety Control by Department of Education, Changsha University of Science and Technology, Changsha 410114, Chinaclkelu@foxmail.com (L.K.); niejie23@126.com (J.N.); 2 School of Civil Engineering, Changsha University of Science and Technology, Changsha 410114, China

**Keywords:** ultra-high performance concrete (UHPC), shear property, shear strength, cast-in-place, steel fiber

## Abstract

In order to study the direct shear properties of ultra-high performance concrete (UHPC) structures, 15 Z-shaped monolithic placement specimens (MPSs) and 12 Z-shaped waterjet treated specimens (WJTSs) were tested to study the shear behavior and failure modes. The effects of steel fiber shape, steel fiber volume fraction and interface treatment on the direct shear properties of UHPC were investigated. The test results demonstrate that the MPSs were reinforced with steel fibers and underwent ductile failure. The ultimate load of the MPS is about 166.9% of the initial cracking load. However, the WJTSs failed in a typical brittle mode. Increasing the fiber volume fraction significantly improves the shear strength, which can reach 24.72 MPa. The steel fiber type has little effect on the shear strength and ductility, while increasing the length of steel fibers improves its ductility and slightly reduces the shear strength. The direct shear strength of the WJTSs made from 16 mm hooked-type steel fibers can reach 9.15 MPa, which is 2.47 times the direct shear strength of the specimens without fibers. Finally, an interaction formula for the shear and compressive strength was proposed on the basis of the experimental results, to predict the shear load-carrying capacity of the cast-in-place UHPC structures.

## 1. Introduction

Shear failure may occur near the geometric discontinuity or joint interfaces of concrete structures, where the cracks are usually perpendicular to the axis of the member, without a bending moment. This shear behavior is known as “direct shear” [1]. Direct shear failure is a sudden and catastrophic failure mode in traditional concrete structures [2]. Although this behavior has been studied on ordinary concrete structures for more than 40 years, it was still not clear if the empirical models can accurately predict the actual shear behaviors [1]. Ultra-high performance concrete (UHPC) is a novel fiber reinforced concrete (FRC) with high strength, excellent service durability and low permeability [3,4,5,6]. It has been extensively used in buildings, bridges and other structural projects with a thin-walled structure [7,8,9]. Thin-walled reinforced concrete structures subjected to a distributed load of short duration (such as explosive loading and seismic load) may not behave plastically at the mid-span and fail there. Some of the beams might fail at positions very close to the support owing to direct shear failure [10]. Hence, it is very important to study the direct shear properties of UHPC structures. Through the numerous experimental studies of FRC [11,12,13], it had turned out that steel fibers have a great effect on the improvement of the shear properties of concrete. Due to the poor interface properties between the coarse aggregates and cements and less fiber volume fraction used in FRC, the shear failures mostly show brittle modes. However, unlike ordinary concrete, FRC and high-strength concrete, UHPC has no coarse aggregates and possesses high compactness, as well as a high fiber volume fraction. Thus, the shear behavior of UHPC may be different from those of traditional concrete, and its shear failure process is worth studying.

Owing to the limitation of the mixing of UHPC, transportation and maintenance ability, structures will inevitably show joint connection problems [14,15,16]. Even in precast UHPC structures, there still remains some components or joints of segments to be cast in situ [17]. Thus, the study of shear properties at joint interfaces is very important for all composite structures [17,18,19,20]. At present, the ultimate limit and serviceability limit state that calculation for traditional concrete structures, because of its low tensile strength, do not need to take into account concrete tensile strength [21,22]. However, the tensile strength of UHPC is very well owing to the bridging effect of continuous steel fibers, and the utilization of the tensile capacity of UHPC has a significant impact on its economic rationality. For segmental precast UHPC structures, current practice is to use multiple key joints that are generally unreinforced and may be dry or epoxied [23,24,25]. For this reason, it could not utilize the beneficial effect of the continuity of steel fiber distribution at the interface and the natural occlusion between the UHPC aggregate matrix well. Hyun-O Jang [26] studied the shear properties of UHPC specimens with Z-shaped specimens. Their results show that the shear strength of the waterjet treated specimens (WJTSs) can reach 32.2% of the monolithic placement specimens (MPSs). However, it is insufficient that each group of specimens contain only one specimen without considering different types of steel fibers. Direct shear performance with different interface treatments for segmental cast-in-place UHPC is worthy of further research.

The objective of this paper is to obtain the failure modes, shear strength and shear slip properties of UHPC in situ wet joints. The variables include fiber types (13 mm straight-type steel fibers (13SSF), 13 mm hooked-type steel fibers (13HSF) and 16 mm hooked-type steel fibers (16HSF)), fiber volume fractions (2.0%, 2.5% and 3.0%) and interface treatment. The direct shear tests were performed on 15 MPSs and 12 WJTSs (flat joints). Besides, in order to evaluate the load-carrying capacity of cast-in-place UHPC structures, an interaction formula with respect to shear and compressive strength and the relative reduction of the shear strength ratio for the WJTSs are offered on the basis of the experimental results.

## 2. Experimental Program

### 2.1. Experimental Specimens

**Figure 1:**
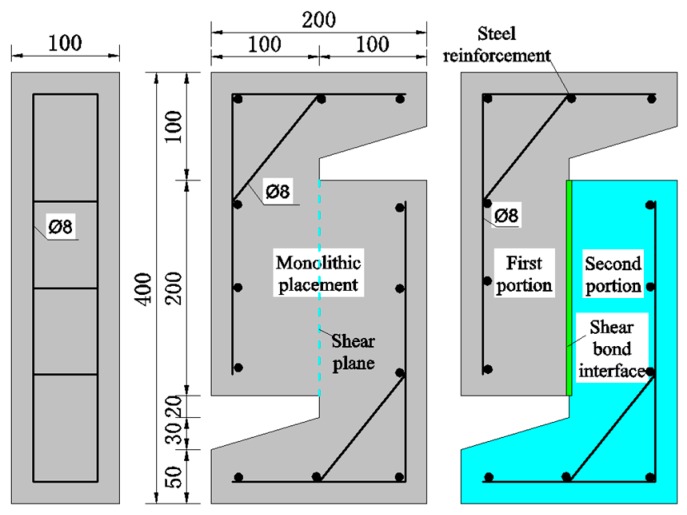
Direct shear test specimen.

Herein, to make the shear transfer of the specimens more consistent with that of the segmental concrete structure, the direct shear test of Z-shaped specimens was used. Dimensions of each specimen are given by 200 mm × 400 mm × 100 mm, in which the shear plane size is 100 mm × 200 mm. In order to avoid the failure of other parts prior to the shear plane, the reinforced bar with a diameter of 8 mm was arranged in these specimens for strengthening. The dimensions for the specimens are illustrated in Figure 1.

The UHPC mixture used in the tests is composed of cementitious material (mix of Portland cement, silica fume and mineral powder), quartz sand and solid polycarboxylate superplasticizer (water reducing efficiency of 30%), and the mix proportion of UHPC is shown in Table 1.

**Figure 2:**
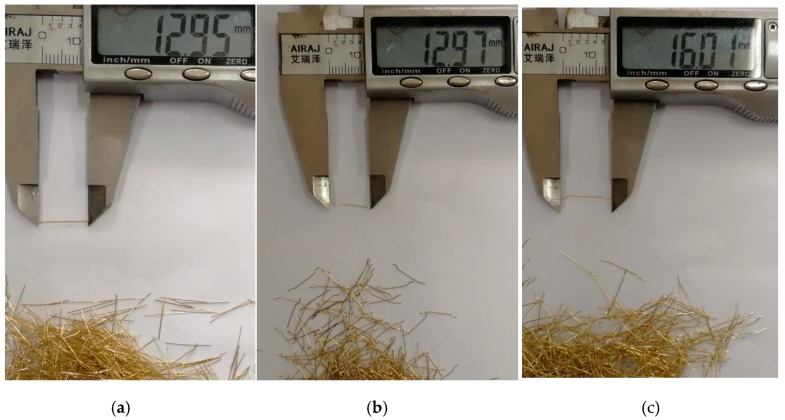
Steel fiber types: (**a**) 13SSF, (**b**) 13HSF and (**c**) 16HSF.

**Figure 3:**
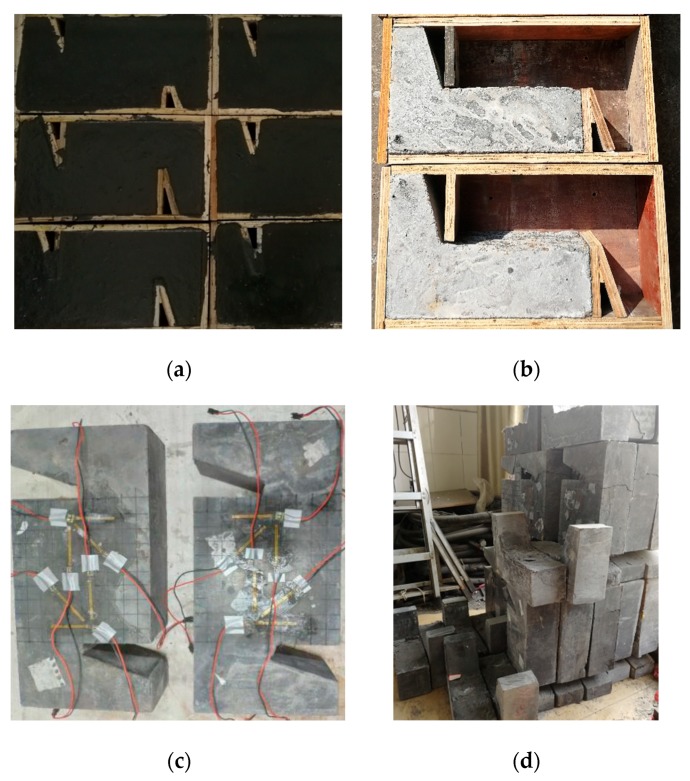
Manufacturing of Z-shaped specimens: (**a**) monolithic placement specimens (MPSs), (**b**) waterjet treated specimens (WJTSs), (**c**) finished specimens after maintenance and (**d**) all completed specimens.

To study the effect of steel fiber types on the shear properties of UHPC structures, three kinds of steel fibers were selected (see Figure 2), namely 13 mm straight-type steel fibers (13SSF), 13 mm hooked-type steel fibers (13HSF) and 16 mm hooked-type steel fibers (16HSF), respectively. The physical and mechanical properties of the steel fibers are shown in Table 2. Besides, three kinds of fiber volume fraction were selected to study the effect of steel fiber volume fraction on the UHPC shear properties, which is 2.0%, 2.5% and 3.0%, respectively (Table 3). In this way, 15 MPSs (Figure 3a) and 12 WJTSs (Figure 3b) were fabricated.

The following steps were conducted to mix the UHPC ingredients:

(1) In a dry mixer (pre-wetting), dry components (cement, silica fume and mineral powder) were added and mixed for 2 min.

(2) Then the mixer was suspended, and fine quartz sand added and stirred for 1 min.

(3) The required solid superplasticizer was poured into the total water outside of the mixer and the solution was added to the mix gradually and stirred for 4 min.

(4) Finally, steel fibers were added manually by slowly sprinkling them into the mixer, to avoid balling and to produce a concrete with uniform material consistency and good workability. Stirring occurred until the steel fibers were well encapsulated and evenly distributed in the slurry. The stirring process lasted about 3 min.

The MPSs were poured at one time and cured by high-temperature steam above 95 ± 3 °C for 48 h after conventional standard curing (temperature 20 ± 2 °C, relative humidity 95%) for two days. In contrast, the preparation of WJTSs appears more complex and follows the procedures below. First, the first portion was poured and cured in the standard environment for two days. Secondly, the shear bond interface of the first portion was treated by the high-pressure waterjet, and they were cured in high-temperature steam condition for 36 h. Then, the second portion was poured and cured in the standard environment for 48 h. Finally, whole specimens, including the first and second portions, were cured in high-temperature steam conditions for 48 h.

**Figure 4:**
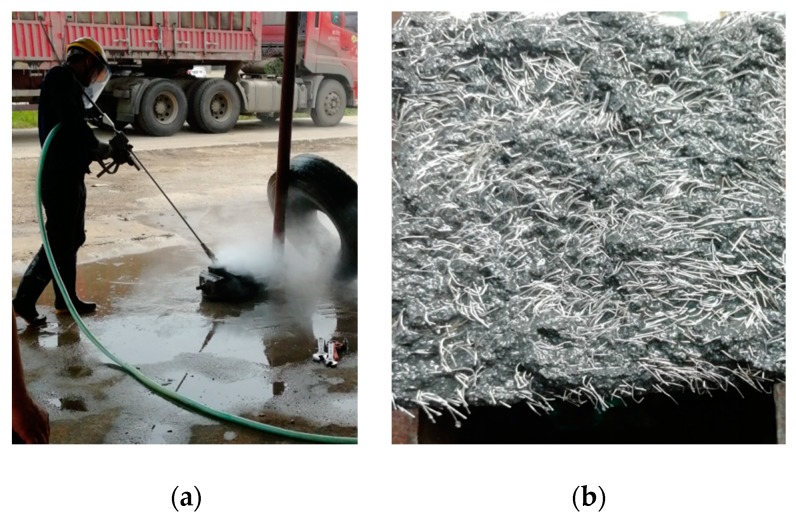
Interface treatment with a high-pressure waterjet. (**a**) Waterjet; (**b**) surface treatment.

In order to utilize the beneficial effect of the steel fibers continuity distribution and the natural occlusion between the UHPC aggregate matrix at the shear plane, the treatment of the shear bond interface needs to ensure the retention of steel fibers and the length of fiber exposure as far as possible. As shown in the Figure 4, the roughing on the concrete surface with a high-pressure waterjet is converging water flow at a point through a high-pressure device and the energy will be greatly weakened once the water flow impacts the specimen surface. Therefore, the process will not cause damage to the inside of the specimen. It is theoretically possible to retain a certain amount of steel fibers at the UHPC shear plane, which has been widely accepted by engineers [26,27].

**Figure 5:**
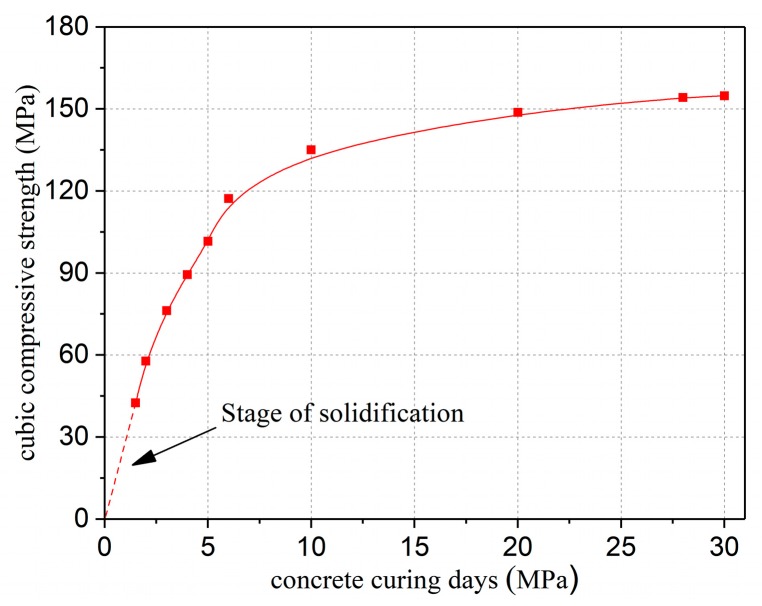
The change in cubic compressive strength of UHPC with curing days under normal temperature.

Figure 5 illustrates the variation of cubic compressive strength of UHPC with curing days under normal temperature. UHPC strength develops rapidly after initial solidification. After 4 days of maintenance, the cubic compressive strength of UHPC is close to 90 MPa. Therefore, in order to ensure the shear bond interface possesses excellent chiseling effect, the specimens should be controlled to chisel after 1.5~4 days of maintenance in practical engineering.

Owing to the existence of a large number of steel fibers at the interface, it is difficult to accurately measure the interface roughness by conventional measurement methods. Thus, the effect of the roughness of the interface is not considered in this experiment. Considering the complexity of steel fiber dispersion, the distribution quantity of steel fiber types is also not considered.

### 2.2. Material Properties

**Figure 6:**
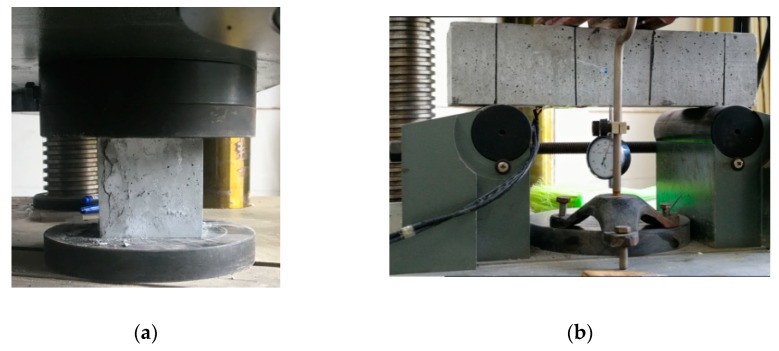
Performance test of UHPC material: (**a**) cubic compression test and (**b**) four-point bending test.

Three cubic compressive specimens (100 mm × 100 mm × 100 mm) and three flexural specimens (100 mm × 100 mm × 400 mm) were prepared to obtain the UHPC material properties. All specimens were cured under the same environment to determine the actual strength of UHPC materials during the test. As illustrated in Figure 6, some material properties experiments of UHPC have been carried out. Table 4 summarizes the material properties of UHPC, including the cubic compressive strength (*f_cu_*) and flexural strength (*f_cf_*).

### 2.3. Loading Process and Measuring Arrangement

**Figure 7:**
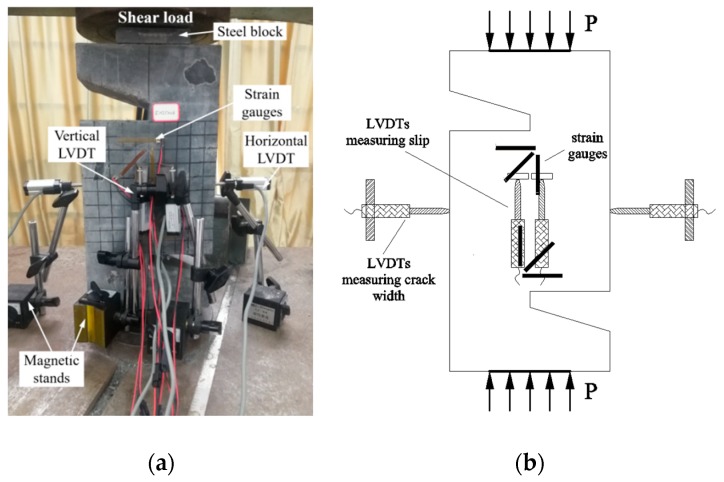
Test set-up. (**a**) Photo of test set-up and (**b**) arrangement of the transducers.

The shear test was conducted on a 2000 kN universal testing machine. In order to examine deformation characteristics, a set of two linear variable differential transducers (LVDTs) was installed on the vertical direction of the specimen to measure the relative deformation under direct shear load at the construction joint. Besides, a set of two LVDTs was arranged at the center of the horizontal shear plane of the specimen for the purpose of measuring the variation of crack width along with the increase of load. To ensure that the LVDTs and strain gauges were fixed firmly as well as the test device was connected reliably, preloading was carried out before formal loading. Besides, when the load-carrying capacity of the specimen drops sharply, the test machine will automatically stop loading. In the process of formal loading, the condition of the crack initiation and extension was observed directly by a high-power magnifier (zoom in 30 times). The test set-up is shown in Figure 7.

After loading, the shear strength of the UHPC specimens under ultimate load can be obtained from Equation (1):

(1) $$\tau =\frac{F_{cr}}{A}$$

where *τ* represents the shear strength (MPa), $F_{cr}$ represents the ultimate load (kN) and *A* represents the shear plane area of specimens (mm^2^).

## 3. Experimental Results and Discussion

### 3.1. Test Results and Analysis of the MPSs

#### 3.1.1. Load-Carrying Capacity and Failure Modes

**Figure 8:**
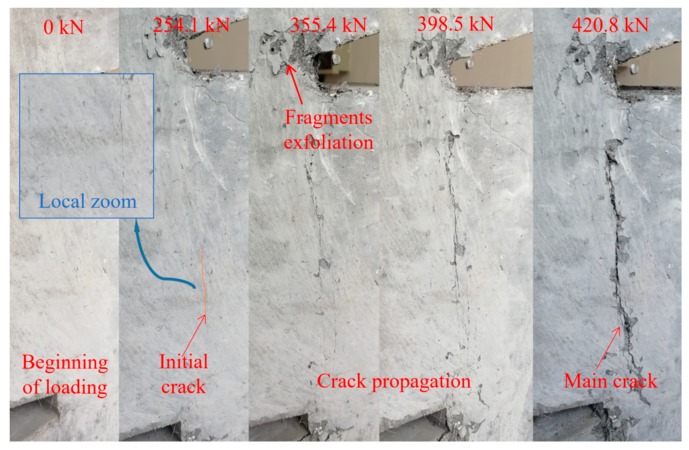
Failure modes and the crack propagation process.

**Figure 9:**
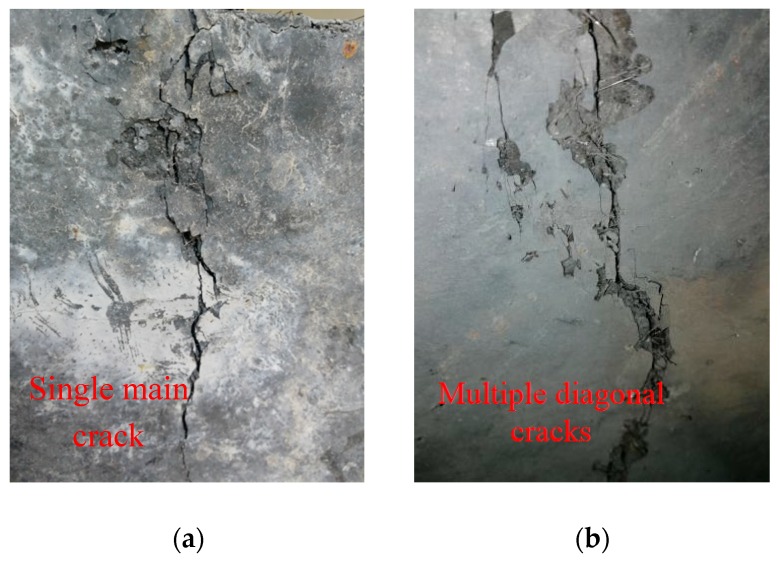
Two typical crack forms: (**a**) single main crack and (**b**) multiple diagonal cracks.

The failure modes of the MPSs are similar. The development of cracks was analyzed based on the MP25H16 specimen as an example. Firstly, there were no changes in the surface of these specimens at the initial loading stage. As the loading continued, the fragments at the shear plane began to exfoliate. After that, the initial cracks appeared on the shear plane and several small cracks appeared instantaneously. Herein, it should be noted that the crack width of 0.05 mm is adopted as the criterion of visible initial cracking [28]. With the increase in loading, fine cracks further spread, connected and penetrated to form a crack zone along the shear failure surface, and the fibers between the crack zones were gradually pulled out or pulled off. Finally, with the further increase in the load, along with a huge sound, specimens were sheared and damaged. The condition of the crack development is shown in Figure 8. In order to verify whether the UHPC specimens still possess the bearing capacity after the main crack occurs, the test machine was restarted to continue loading. It turns out the load could still reach 1/2~2/3 of the ultimate load. On the basis of the testing results, it can be seen that there are two main crack modes in the failure modes for these specimens, namely the single main crack (Figure 9a) and multiple diagonal cracks (Figure 9b), respectively.

**Figure 10:**
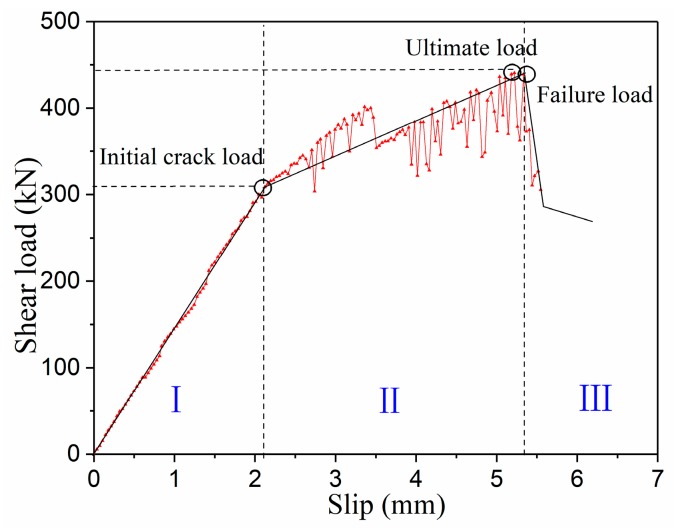
UHPC shear load–slip curve.

Since the failure characteristics of the specimens are similar, we took the MP25H13 specimen as an example to describe the shear load–slip relationship of the MPSs. The shear load–slip curve of MP25H13 specimen is shown in Figure 10. The results show that the direct shear cracking failure characteristics of UHPC can be divided into three domains. The first domain is a linear development stage in which the relationship between load and slip is basically linear (ideal status). The second domain is a crack growth stage. With the initial stiffness degenerating, the slope of the load–slip curve begins to decline. After the micro-crack occurs, the stiffness gradually decreases, and then the load enters a linear deviation domain. As a whole, because the bridging action of the steel fibers suppresses the crack expansion, the load increases in the alternation as the slip increases. The third domain is the shear failure stage. At this stage, large amounts of steel fibers are broken or pulled out from the matrix, and the specimens are rapidly destroyed.

#### 3.1.2. Shear Strength Results

For the MPSs, the baroclinic bar and the steel fibers passing through the shear plane act together to form a truss to resist the shear force along the shear plane. The effect of steel fibers on the shear properties of UHPC was analyzed, and the test results of fifteen MPSs are listed in Table 5.

**Figure 11:**
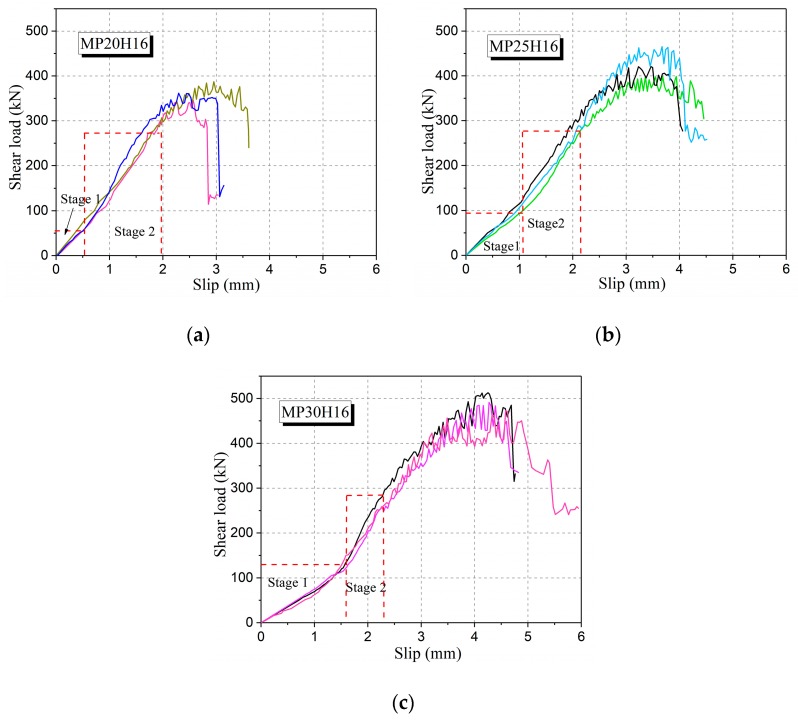
Shear load–slip curves of UHPC specimens with a different fiber volume fraction: (**a**) MP20H16, (**b**) MP25H16 and (**c**) MP30H16.

According to Table 5, the maximum shear strength of UHPC can reach 24.72 MPa (MP30H16), which is 1.37 times the shear strength of MP20H16. It can be seen that the direct shear strength of UHPC increases with the increase of the fiber volume fractions. In addition, the shear strength of MP25H16 is 19.6% higher than that of MP20H16, while the shear strength of MP30H16 is only 14.8% higher than that of MP25H16. This shows that the shear strength of UHPC increases more obvious at the initial phase of the fiber volume fraction increment. According to the ratio of ultimate load to initial crack load of the specimens and shear slip relationship along the shear plane under different fiber volume fraction (Figure 11), when the fiber volume fraction is fewer, cracking failure characteristics of UHPC are close to brittle failure. Based on the above analysis, it can be considered that the ductile characteristic and direct shear strength of the monolithic placement specimens with appropriate steel fibers can be improved in direct shear load.

As shown in Figure 11, the initial stiffness of the specimens (Stage 1) is reduced with the increase in steel fibers. This can be explained as follows. On the one hand, the uniformity of fiber mixing will be affected with the increase of steel fiber content, and the probability of internal defects (voids) of the UHPC will also be increased. On the other hand, the shear stiffness at the early stage of loading is predominantly dependent on the overall modulus of the matrix. Only after micro-cracks occur, the shear force will be balanced by the steel fibers and the matrix. Thus, with the increase in steel fiber content, the overall modulus of the matrix itself is reduced, leading to a reduced shear stiffness at Stage 1. With the further increase in load, micro-cracks occur, and the influence of the internal defects is gradually eliminated. The subsequent stiffness (Stage 2) mainly depends on the comprehensive modulus of the steel fiber and matrix. It should be noted that these phenomena are only reflected when the fiber content is more than 2.5%.

**Figure 12:**
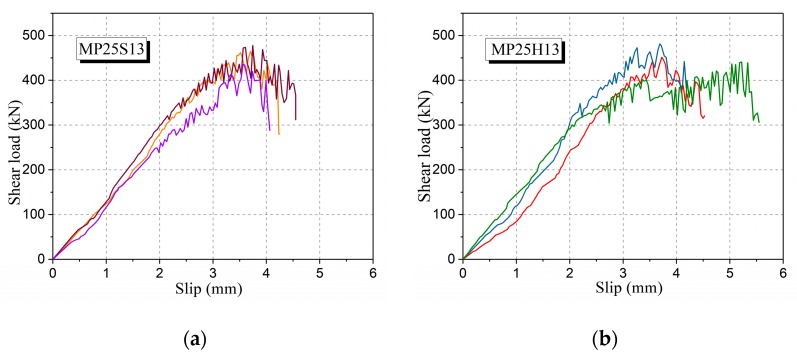
Shear load–slip curves of UHPC specimens with different fiber types: (**a**) MP25S13 and (**b**) MP25H13.

Table 5 also shows the effect of different types of fibers on the UHPC shear strength. Under the same volume fraction of fibers (2.5%), the shear strengths of MP25S13, MP25H13 and MP25H16 are almost at the same level. It shows that the shear strength is not significantly influenced by the shape of the steel fibers. In the case of a certain fiber length, whether or not the hooked-type steel fiber is used has little effect on the shear strength of UHPC. However, when using the same shape fibers, increasing the fiber length will slightly reduce the shear strength of UHPC. This is because the UHPC specimens with short fibers possess more fibers at the same volume fraction. From the previous analysis, increasing the fiber numbers is an important way to enhance the compressive strength of UHPC. In addition, according to the ratio of ultimate load to the initial crack load of the specimens and shear load–slip curves under different fiber types (Figure 12), the use of hooked-type fibers has little effect on the ductile failure characteristics of UHPC specimens.

### 3.2. Test Results and Analysis of the WJTSs

#### 3.2.1. Load-Carrying Capacity and Failure Modes

**Figure 13:**
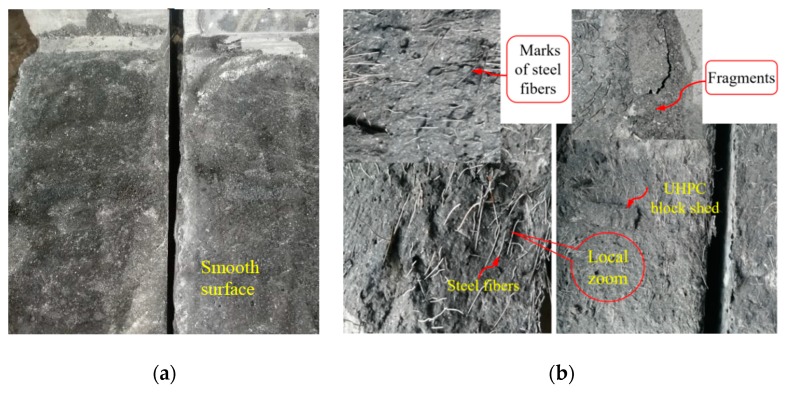
Direct shear failure of cast-in-place specimens: (**a**) without steel fibers and (**b**) doped with steel fibers.

The shear failure characteristic of the WJTSs is a typical brittle failure, which occurs abruptly and without obvious symptoms. Similarly, the shear failure of the specimens is accompanied by a loud noise, especially for the WJ25H16, while the sound of the specimens without steel fibers will not be harsh. From the failure of the bond interface of the specimens, the failure surface of the specimens without fibers is relatively smooth (Figure 13a). But parts of the poured UHPC are embedded together successively, and the peeling phenomenon between the UHPC blocks interfaces is not obvious. The bonding effect of steel fiber reinforced specimens (especially for the WJ25H16) is excellent due to the bridging effect of steel fibers (Figure 13b), which is similar to the MPSs. When the specimens are damaged, obvious fiber pullout marks and UHPC fragments can be seen. Thus, steel fibers can play an important role in enhancing the shear plane strength for the WJTSs.

#### 3.2.2. Shear Strength Results

Because of the uneven surface of the UHPC flat joint interface, the interface subjected to shear force can provide resistance through the friction between the poured aggregates at the interface. When the applied load increases, some UHPC aggregates are sheared off, which results in a decrease in shear stiffness and rapid deformation of the interface. Therefore, the shear failure of the specimen without fibers occurs directly once the aggregates are crushed. The test results of 12 WJTSs are given in Table 6.

**Figure 14:**
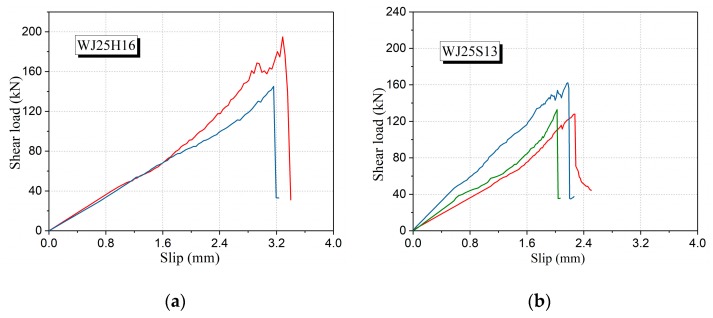

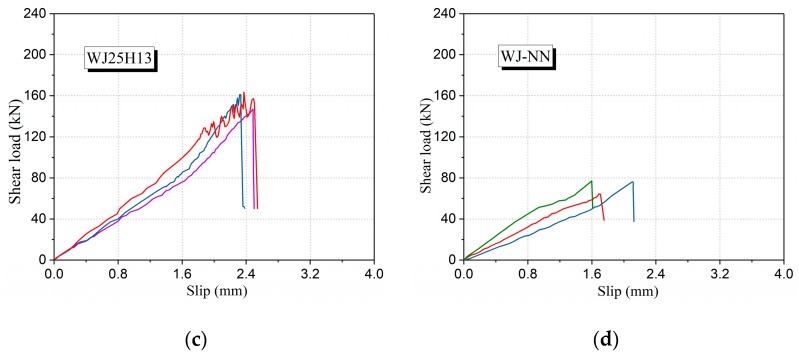
Shear load–slip curves of UHPC specimens with different fiber types: (**a**) WJ25H16, (**b**) WJ25S13, (**c**) WJ25H13 and (**d**) WJ-NN.

From Table 6 as well as the shear load–slip curves of UHPC specimens with different fiber types (Figure 14), the cracking load of the specimens without fibers is very close to the failure load, and it is difficult to accurately distinguish the cracking load from the failure load. The difference is that the ratio of initial crack load to ultimate load of WJ25H16 is significantly greater than that of other conditions. Besides, the direct shear strength of WJ25H16 can reach 9.15 MPa, which is 2.47 times that of WJ-NN and 1.29 times that of WJ25S13, respectively. Thus, increasing the length of the fibers and using profiled fibers can significantly improve the interfacial bonding force. From the above analysis, it can be seen that the interface shear strength of UHPC can be significantly increased by using 16HSF.

## 4. Analytical Study

### 4.1. Interaction of Compressive and Shear Strength of the MPSs

The experimental results demonstrate that the influence of concrete strength should be considered when calculating the ultimate shear strength of the MPSs without pre-cracking. The experimental data are shown in the Table 7. Considering that the shear strength and shear failure characteristics of UHPC are closely related to the compressive strength of concrete and the bridging effect of steel fibers [29], the relationship between the compressive strength and shear strength of concrete is proposed based on the test results, as shown in Equation (2).

Owning to the orientation of the steel fibers inside the concrete matrix, it is affected by a number of parameters, which are essentially the geometry of the fibers and their interaction effects (fibers–aggregates–formwork), the flowability of the concrete, the means of pouring and compacting of the concrete [12]. In addition, the distribution and orientation of fibers is, in turn, the parameter which most influences the ductility of UHPC. Hence, Equation (2) draws into the influence coefficient of steel fiber dispersion *β_cr_* on direct shear bearing capacity to weaken the above effects.

(2) $$\tau_{mn}=(1+\beta_{cr}\lambda_{f})\sqrt{f_{cu}/0.45},$$

(3) $$\lambda_{f}=\frac{\rho_{f}l_{f}}{d_{f}},$$

where $\tau_{mn}$ is the shear strength of the MPSs, $\rho_{f}$ is the volume fraction of steel fibers, $l_{f}/d_{f}$ stands for the ratio of length-diameter of steel fibers, $\lambda_{f}$ is the characteristic coefficient of steel fibers, and $\beta_{cr}$ is the influence coefficient of steel fiber dispersion; the experimental value of 0.1 was chosen in this paper.

As can be seen from Table 7, the variations between the calculated shear strength and the experimental shear strength are insignificant, in which the mean value of $\tau_{exp}$**/**$\tau_{cal}$ is 0.986 and the difference is only 0.014. For this reason, Equation (2) can provide a reference for the design of cast-in-place UHPC structures. It is worth mentioning that the configuration of shear reinforcement is not considered in this paper. Therefore, Equation (2) is only applicable to the condition of no shear reinforcement, while other situations, including the vertical shear stress direction of steel bar arrangement, need to be further studied.

### 4.2. Relative Reduction of Shear Strength Ratio of the WJTSs

**Figure 15:**
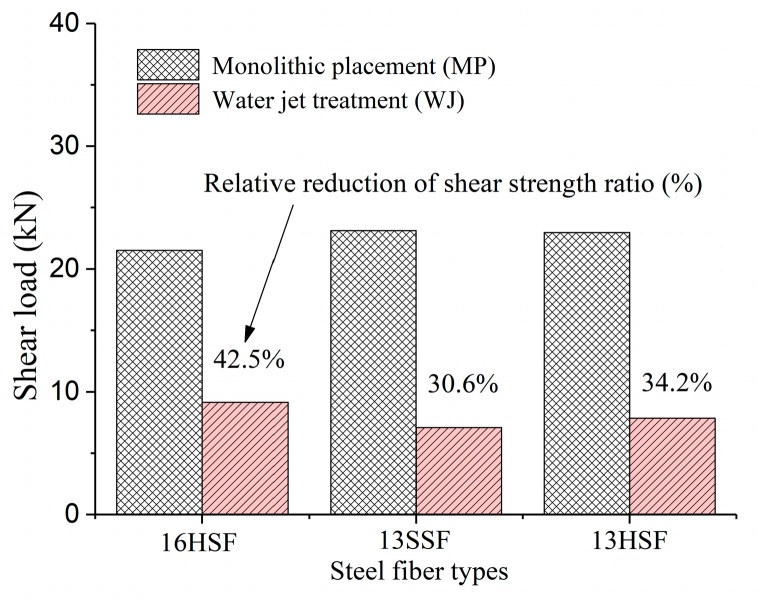
Relative reduction of the shear strength ratio with different fiber types.

Figure 15 shows the relative reduction of the shear strength ratio with different fiber types. Compared with the MPSs, the shear strength of WJ25H16 can reach 42.5% of MP25H16, that of WJ25S13 can reach 30.6% of MP25S13, and that of WJ25H13 can reach 34.2% of MP25H13. Therefore, the maximum shear strength of the UHPC shear bond interface treated by a waterjet can reach 42.5% of the monolithic placement. It can be concluded that the shear strength at the interface of the UHPC specimens reinforced with steel fibers (because of the contribution of the aggregate biting force, interface friction force and steel fiber drawing force) can be effectively utilized under the appropriate interface treatment. What is insufficient is that the number of specimens in this study is still fewer, and the study of direct shear strength under a different fiber volume fraction has not been carried out, so the relative reduction formula of the shear strength ratio under various conditions cannot be obtained accurately.

## 5. Conclusions

This study investigates the direct shear strength and failure mechanism of Z-shaped specimens through the push-off test. A total of 27 specimens with the test parameters of steel fiber shape, steel fiber volume fraction and interface treatment, were designed to test their shear strength, load-carrying capacity and failure modes. Based on the testing results, the following conclusions can be drawn:

(1) Ductile characteristics of the monolithic placement specimens with appropriate steel fibers can be improved in direct shear load, and the ultimate load can reach 166.9% of the initial cracking load.

(2) Increasing the steel fiber volume fraction can significantly improve the shear strength of UHPC structure. Direct shear strength of UHPC specimens with 3.0% volume fraction can reach 24.72 MPa. In addition, the steel fiber shape has little effect on the shear strength and ductility, while increasing the length of steel fibers improves its ductility and slightly reduces the shear strength.

(3) The waterjet treatment for the interface of the adjacent segment is an effective way to improve the direct shear performance of cast-in-place segmental UHPC structures. The direct shear strength of the specimens with the waterjet treatment can reach 42.5% of the monolithic placement specimens.

(4) The formula proposed for predicting the direct shear strength of the cast-in-place UHPC structures shows good agreement with the test results.

Although the feasibility of the segmental cast-in-place UHPC structure has been validated and the influence of steel fiber on shear performance has been obtained through the experimental studies in this paper, further experimental studies are still needed, including the configuration of shear reinforcement at the interface, the type of key joints at the interface (shape, structure size), the number of key joints, the level of normal stress and the roughness of the interface.

## Figures and Tables

**Table 1:** Mix proportion of the ultra-high performance concrete (UHPC; mass).

Cement	Quartz Sand	Cementitious Material	Water	Superplasticizer
1.00	1.00	1.690	0.304	0.041

**Table 2:** Physical and mechanical properties of the steel fibers.

Types	Density (g·cm^−3^)	Length (mm)	Diameter (mm)	Ratios of Length–Diameter	Modulus of Elasticity (GPa)	Tensile Strength (MPa)	External Features
13SSF	7.8	13	0.2	65	205	2850	straight-type
13HSF	7.8	13	0.2	65	205	2850	hooked-type
16HSF	7.8	16	0.2	80	205	2850	hooked-type

**Table 3:** Shear specimen parameters.

Specimen Type	Specimen Number	Steel Fiber Volume Fraction	Types of Steel Fibers
MPSs	MP25S13	2.5%	13SSF
	MP25H13	2.5%	13HSF
	MP20H16	2.0%	16HSF
	MP25H16	2.5%	16HSF
	MP30H16	3.0%	16HSF
WJTSs	WJ25S13	2.5%	13SSF
	WJ25H13	2.5%	13HSF
	WJ25H16	2.5%	16HSF
	WJ-NN	0	/

Number Description: “MP” stands for the condition of monolithic placement, “WJ” stands for the condition of waterjet treatment; “20”, “25” and “30” stands for the volume fraction of steel fibers; “H” stands for the hooked-type fibers, “S” stands for the straight-type fibers; “13” and “16” stands for the length of the fibers; and “NN” stands for non-doped fibers.

**Table 4:** Test results of the UHPC materials (unit: MPa).

Specimens	Cubic Compressive Strength (*f_cu_*)	Average Cubic Compressive Strength (*f_cu_*)	Coefficient of Variation	Flexural Strength (*f_cf_*)	Average Flexural Strength (*f_cf_*)	Coefficient of Variation
MN25S13	163.90	165.50	0.010	36.92	38.75	0.036
	164.72			39.09		
	167.89			40.25		
MN25H13	164.10	161.43	0.012	35.50	36.43	0.033
	160.77			35.68		
	159.42			38.11		
MN20H16	152.21	150.25	0.011	31.78	32.81	0.035
	150.38			32.24		
	148.15			34.42		
MN25H16	158.24	159.35	0.023	44.52	40.49	0.070
	155.60			38.45		
	164.21			38.51		
MN30H16	184.38	178.50	0.026	42.17	42.66	0.077
	173.12			46.89		
	178.01			38.92		

**Table 5:** Direct shear test results of the MPSs.

Specimen Number	Initial Crack Load $F_{ci}$/kN	Ultimate Load $F_{cr}$/kN	Ultimate Load $F_{cr}$/kN (Average)	Shear Strength *τ*/MPa	Shear Strength *τ*/MPa (Average)	Coefficient of Variation	$F_{cr}$/$F_{ci}$	$F_{cr}/F_{ci}$ (Average)
MP20H16	262.2	340.7	360.1	17.04	18.01	0.055	1.299	1.296
	294.3	387.5		19.38			1.317	
	276.6	352.0		17.60			1.273	
MP25H16	278.6	405.6	430.6	20.28	21.53	0.059	1.456	1.604
	254.1	420.8		21.04			1.656	
	273.8	465.5		23.28			1.700	
MP30H16	286.4	515.6	494.5	25.78	24.72	0.035	1.800	1.669
	296.4	494.8		24.74			1.669	
	307.3	473.0		23.65			1.539	
MP25S13	291.0	469.8	462.7	23.49	23.13	0.028	1.614	1.560
	307.2	473.6		23.68			1.542	
	291.5	444.6		22.23			1.525	
MP25H13	284.2	454.3	459.8	22.72	22.98	0.035	1.599	1.630
	261.3	481.5		24.08			1.843	
	306.2	443.5		22.18			1.447	

**Table 6:** Direct shear test results of the WJTSs.

Specimen Number	Initial Crack Load $F_{ci}$/kN	Ultimate Load $F_{cr}$/kN	Ultimate Load $F_{cr}$/kN (Average)	Shear Strength *τ*/MPa	Shear Strength *τ*/MPa (Average)	Coefficient of Variation	$F_{cr}$/$F_{ci}$	$F_{cr}$/$F_{ci}$ (Average)
WJ25H16	159.7	205.60	182.9	10.28	9.15	0.139	1.287	1.188
	160.5	195.40		9.77			1.217	
	139.5	147.75		7.39			1.059	
WJ25S13	115.9	128.4	141.8	6.42	7.09	0.110	1.108	1.042
	154.9	163.7		8.18			1.057	
	129.3	133.2		6.66			1.030	
WJ25H13	141.2	146.9	157.1	7.35	7.86	0.046	1.040	1.153
	140.1	161.3		8.06			1.151	
	128.8	163.2		8.16			1.267	
WJ-NN	67.2	70.2	74.1	3.51	3.71	0.039	1.045	1.040
	70.0	75.2		3.76			1.074	
	77.0	77.0		3.85			1.000	

**Table 7:** Tests of UHPC shear specimens.

Specimen Number	Ratios of Length-Diameter $l_{f}/d_{f}$	Volume Fraction of Fibers $\rho_{f}$	Characteristic Coefficient of Steel Fibers $\lambda_{f}$	Shear Strength $\tau_{exp}$/MPa	Cubic Compressive Strength $f_{cu}$/MPa	Shear Strength $\tau_{cal}$/MPa	$\tau_{exp}$/$\tau_{cal}$
MP20H16	80	2.0%	1.6	18.01	150.25	20.11	0.90
MP25H16	80	2.5%	2.0	21.53	159.35	22.58	0.95
MP30H16	80	3.0%	2.4	24.72	178.50	24.69	1.00
MP25H13	65	2.5%	1.625	22.98	161.43	22.02	1.04
MP25S13	65	2.5%	1.625	23.13	165.50	22.29	1.04
